# Structural insights into conformational stability of both wild-type and mutant EZH2 receptor

**DOI:** 10.1038/srep34984

**Published:** 2016-10-07

**Authors:** Imlimaong Aier, Pritish Kumar Varadwaj, Utkarsh Raj

**Affiliations:** 1 Department of Bioinformatics, Indian Institute of Information Technology Allahabad, Uttar Pradesh, India

## Abstract

Polycomb group (PcG) proteins have been observed to maintain the pattern of histone by methylation of the histone tail responsible for the gene expression in various cellular processes, of which enhancer of zeste homolog 2 (EZH2) acts as tumor suppressor. Overexpression of EZH2 results in hyper activation found in a variety of cancer. Point mutation on two important residues were induced and the results were compared between the wild type and mutant EZH2. The mutation of Y641 and A677 present in the active region of the protein alters the interaction of the top ranked compound with the newly modeled binding groove of the SET domain, giving a GLIDE score of −12.26 kcal/mol, better than that of the wild type at −11.664 kcal/mol. In depth analysis were carried out for understanding the underlying molecular mechanism using techniques viz. molecular dynamics, principal component analysis, residue interaction network and free energy landscape analysis, which showed that the mutated residues changed the overall conformation of the system along with the residue-residue interaction network. The insight from this study could be of great relevance while designing new compounds for EZH2 enzyme inhibition and the effect of mutation on the overall binding mechanism of the system.

Enhancer of zeste homolog protein 2 (EZH2) is the catalytic subunit of a protein complex called the polycomb repressive complex 2 (PRC2). PRC2 has been identified in a wide range of organisms in the form of chromatin modifiers which are conserved in nature. PRC2 consists of five subunits in humans, namely EZH2, EED, SUZ12, RbAp46/48, and AEBP2. The mono-, di- and trimethylation of Lysine 27 of histone H3 (H3K27) of chromatin is carried out by EZH2, which also acts as a transcriptional repressor and an epigenetic marker. Trimethylation of the histone molecule is carried out by an enzyme that catalyzes the reaction and this catalyzation process is carried out via the SET (Su (var) 3–9, Enhancer-of-zeste and Trithorax) domain, which is a conserved feature present in EZH2. It has been found that the over expression of EZH2 leads to a number of cancer with elevated levels of EZH2 found in breast as well as prostate cancer patients. The elevated level of EZH2 may be attributed to the mutation of residues Y641 to a phenylalanine (Y641F) and A677 to a glycine (A677G) present in the SET domain, which increases the activity of trimethylation in the protein^1^,^2^.

Over the years, potent chemically synthesized inhibitors for EZH2 have been developed, such as GSK126^3^, EPZ005687^4^, El1^5^ and UNC1999^6^. Although chemically synthesized drugs are often found to be potent enough to be effective in low dosage, they are associated with various side effects, which can be verified through a series of clinical trials and patient history. Moreover, synthetic compounds are not found in nature and have to be synthesized in the laboratory, which is not so in the case of naturally occurring compounds. Several drugs available in the market today contain some form of natural products or their derivatives, many of which are microbial, fungi or plant based. The compounds isolated from natural sources can also be used as templates for creating more potent lead molecules.

The natural product library screened against the said target consists of well-known molecules which has proven anticancer and antiviral effect and being used in human population. Hence it can be safely assumed that the screened compound will have less side effect and can be administered without long and delayed phase of clinical trials. The number of potential drug-like candidates present in nature that are yet to be discovered are nearly limitless. By isolating these natural compounds, it may be possible to come up with more potent drugs which can further be manipulated so as to make it more efficient and safe for humans.

Literature survey shows that the active region of the structure of EZH2 enzyme, which is present in the SET domain, does not display ligand docking despite efforts being made in order to crystallize the structure in the presence of cofactors and inhibitors^7^. This can be solved by remodeling the active site region of the enzyme by selecting the best homologous structure available. By remodeling and refining the 3D structure of the enzyme, not only can we provide better docking results, but can also determine the binding mechanism of ligands to the active site. Insights to the effect of mutation on the mechanism of binding can also be further understood, thus giving a clear overview of all the underlying process on a molecular level.

## Results

### Structure of the target protein

**Figure 1:**
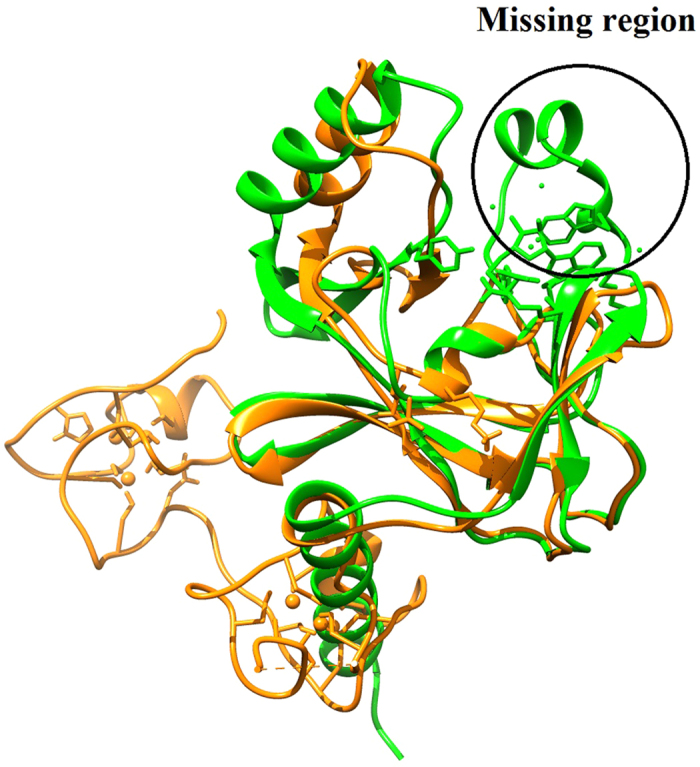
Superposition of EZH2 with hSET8 SET domain. The 3D structure of EZH2 (PDB ID.4MI5) represented in orange superimposed against hSET8 (PDB ID: 1ZKK), while the missing active site region is marked by a circle.

The 3D structure of EZH2 containing 229 amino acid residues was determined by X-Ray crystallography at a resolution of 2.0 Å. A CXC domain was observed in the C-terminal region of EZH2 along with catalytic I-SET, SET, and post-SET domains. The residues in the active site of the SET domain were partially missing and hence the EZH2 PDB model was considered unsuitable for further molecular docking studies leading to the remodeling of the protein. The active site SET domain of the protein, recreated using Modeller for both the wild type as well as the mutant, showed a binding groove which was otherwise noticeably absent in the original structure (Fig. 1).

### Screening and molecular docking analysis

**Figure 2:**
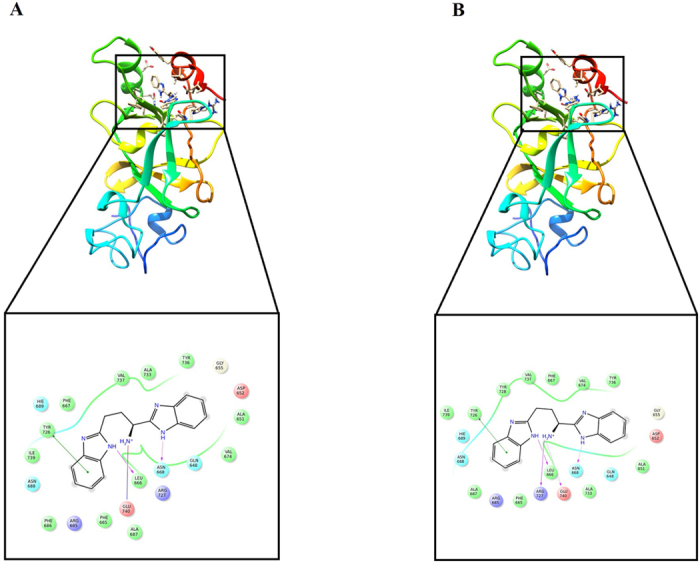
Docking of compound STOCK1N-05528 to the active site of EZH2. Structure of (**A**) Wild type and (**B**) Mutant with ligand interaction between STOCK1N-05528. Both the mutant and the wild type shows pi-pi stacking with Y726. However, mutant variant forms an extra salt bridge with the backbone of residue R727 and a hydrogen bond with the side chain of residue E740.

Docking studies showed interactions of the top ranked compound with several residues on the receptors of both the wild type and the mutant protein (Fig. 2). On the basis of glide G-score obtained from extra precision docking, compound ID STOCK1N-05528, also known as 1, 3-Bis (1H-benzimidazol-2-yl) propan-1-amine, was found to be more effective against both wild type as well as mutant protein receptor as compared to the reported inhibitors as depicted in Table 1. There is also evident interaction of the ligand with at least two amino acid residues in the active region of the protein (Y726 and F667).

### Molecular dynamics simulation analysis

Molecular dynamics simulation is a method carried out to find out the movement of atoms and molecules over a given period of time. The energy of the top ranked complex carried out by molecular dynamics simulation was found to be −84978.25 kcal/mol for the wild type, while the mutant showed an energy of −84992.23 kcal/mol.

**Figure 3:**
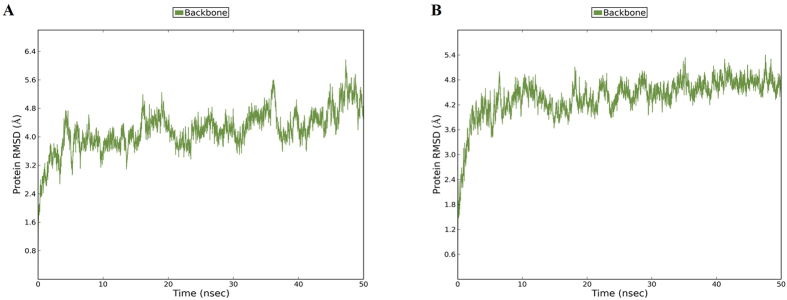
RMSD graphs for the backbone of EZH2 for two systems. Plot of RMSD for (**A**) Wild type and (**B**) Mutant for 50 ns of simulation. The molecular dynamics simulation for wild type system shows a gradual increase in the RMSD value with fluctuations, stabilizing at an average of 4.2 Å, while the mutant variant shows a sudden increase in RMSD value with less fluctuations, stabilizing at an average of 4.3 Å.

Root mean square deviation (RMSD) is used for measuring the difference between the backbones of a protein from its initial structural conformation to its final position. The stability of the protein relative to its conformation can be determined by the deviations produced during the course of its simulation. The smaller the deviations, the more stable the protein structure. RMSD value for the C-alpha backbone was calculated for 50 ns simulation in order to check for the stability of both the systems. From the RMSD simulation shown in Fig. 3, it can be observed that the wild type system equilibrated after 5 ns, however fluctuations were visible over the course of simulation with the structure maintaining at a level of 4.2 Å after 10 ns. In the case of the mutant variant, equilibration was obtained at around 8 ns and the system remained stable after 10 ns at 4.3 Å till the end of the simulation (Fig. 3). The RMSD simulation showed that the mutant maintained an overall stability throughout 50 ns of simulation while the wild type displayed more fluctuations.

**Figure 4:**
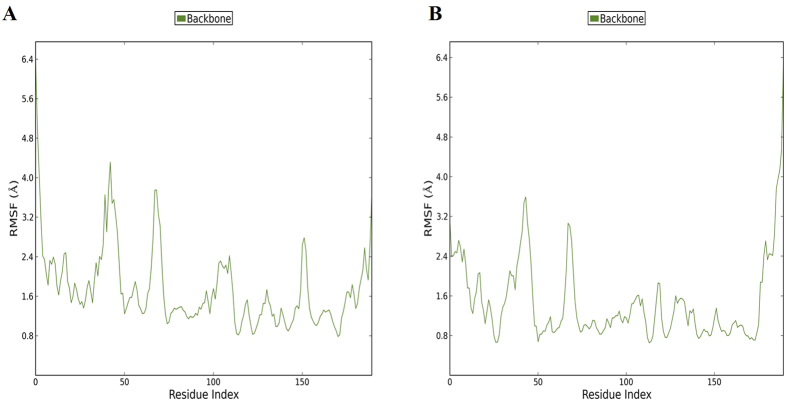
RMSF graphs for the backbone of EZH2 for two systems. Plot of RMSF for (**A**) Wild type and (**B**) mutant for 50 ns of simulation. The C-alpha backbone for wild type EZH2 shows more fluctuation at residue index 150. The same peak for the mutant variant is significantly lower, indicating less motion in the mutant.

Protein regions displaying higher levels of flexibility were calculated. The residue-based root mean square deviation (RMSF) of the backbone for the wild type system displayed more flexible residues at residue number 100 to 110 as compared to the mutant EZH2 system. Also the peak at residue 150 is more defined in the wild type indicating more movement (Fig. 4).

### Residue interaction network analysis

**Figure 5:**
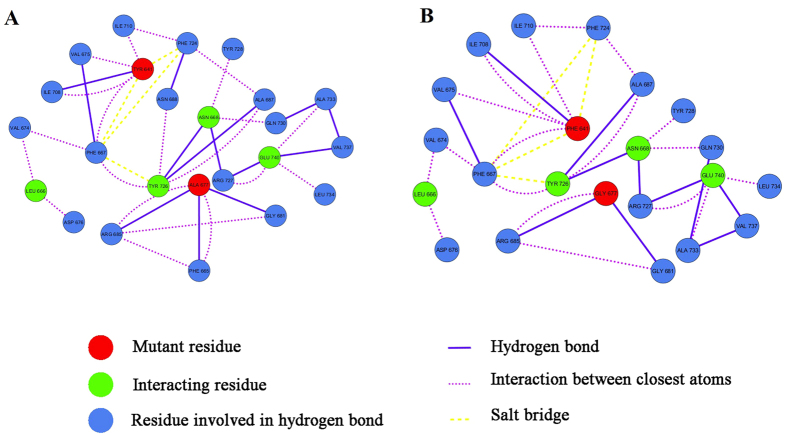
Residue interaction networks showing the interactions between important residues. Comparison of Residue interaction networks (RIN) between (**A**) wild type and (**B**) mutant system. Residue G677 of mutant variant is missing an interaction with residue F665 which is otherwise present in the wild type in close association with the ligand.

Residue interaction network analysis is a strategy for identifying key residue interactions and can be used to point out the differences between wild type and mutant systems. The relationship between key residues of the wild type and the F641 and G677 mutant were made using residue interaction network. Analyzing the RIN plot showed that the mutant residues changed the interaction of the network when compared to the wild type. In the wild type, the residue A677 forms a hydrogen bond with residue F665 whereas there is no interaction of G677 with F665 in the case of the mutated system (Fig. 5).

### Principle component analysis (PCA)

**Figure 6:**
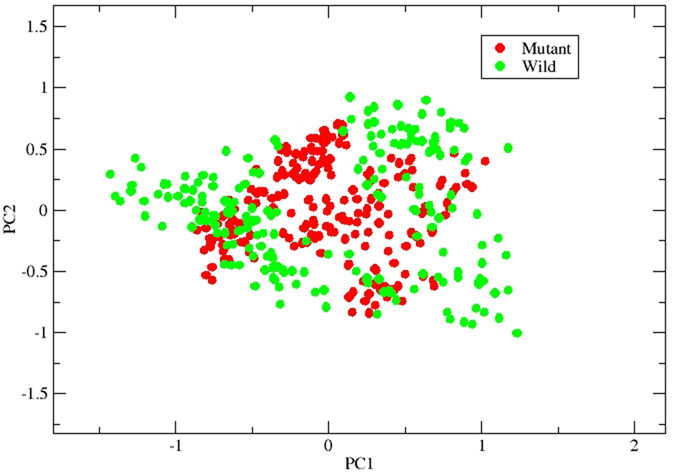
PCA scatter plot as determined by ProDy. PCA scatter plot along first two principal components, PC1 and PC2 showing difference between the wild type and mutant systems.

**Figure 7:**
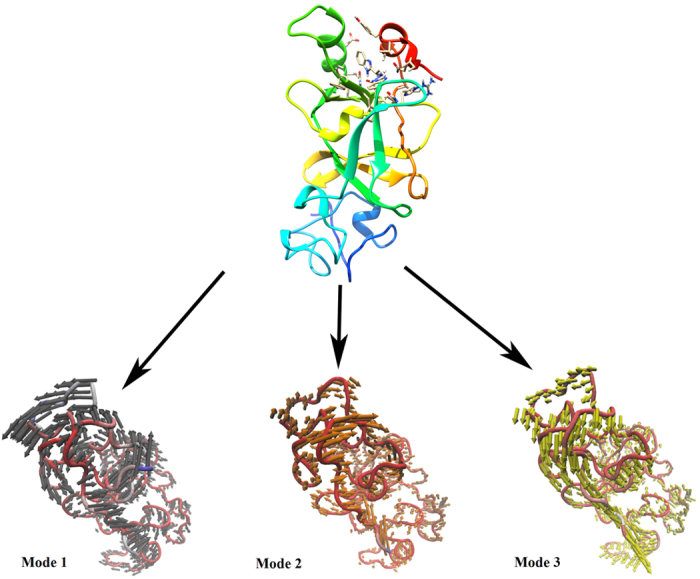
Motion of three different modes of wild type protein depicted by porcupine plot. Porcupine plot showing motion related to wild type complex bound to STOCK1N-05528. Black, orange and yellow arrows indicate motion along modes 1, 2 and 3 respectively.

**Figure 8:**
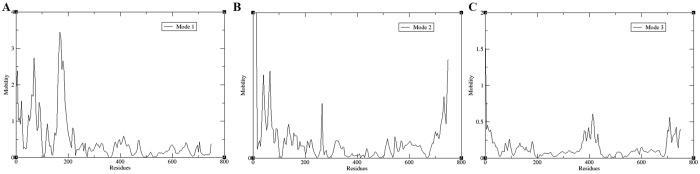
Residue based mobility plot showing the mobility of wild type EZH2 bound to STOCK1N-05528 across different modes.

**Figure 9:**
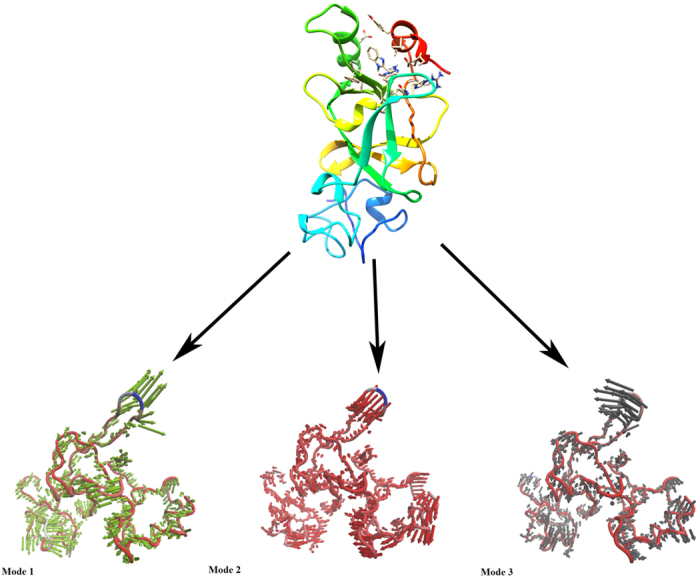
Motion of three different modes of mutant protein depicted by porcupine plot. Porcupine plot showing motion related to mutant complex bound to STOCK1N-05528. Green, red and black arrows indicate motion along modes 1, 2 and 3 respectively.

**Figure 10:**
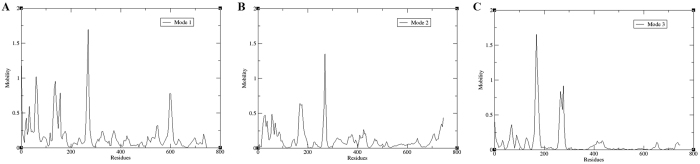
Residue based mobility plot showing the mobility of wild type EZH2 bound to STOCK1N-05528 across different modes.

The scatter plot generated by the wild type and the mutant as shown in Fig. 6 indicated a significant difference between the two systems. The overall motion between the two systems, as shown by the plot, is varied. Major motion of proteins along specific directions represented by eigenvectors can be shown by normal modes, for which porcupine plots were selected, each with three low frequency modes for visualizing the difference in motion between the two systems. It was observed that for the wild type, majority of the motion were due to the contribution of residues number 5, 69, 169 and 269 across all three modes as shown in Figs 7 and 8, while for the mutant, residues number 61, 137, 169, 269 and 597 played a significant role in the contribution of motion across three modes as depicted in Figs 9 and 10.

**Figure 11:**
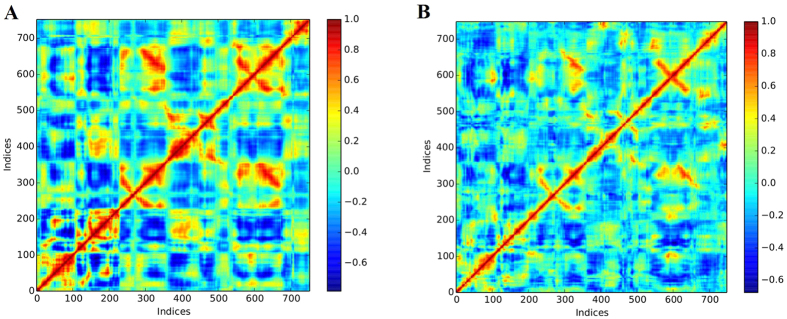
Comparison of cross correlation matrices of wild type and mutant systems. Cross correlation matrix of C-alpha atoms during 50 ns simulation for (**A**) wild type and (**B**) mutant. The range of motion is indicated by various colors in the panel. Red indicates positive correlation whereas blue indicates anticorrelation.

Cross-correlation matrix of the C-alpha displacement indicated complex correlated and anti-correlated motions in the wild type system while the mutant system indicated mostly anti-correlated motion as shown in Fig. 11.

### Free energy landscape (FEL)

**Figure 12:**
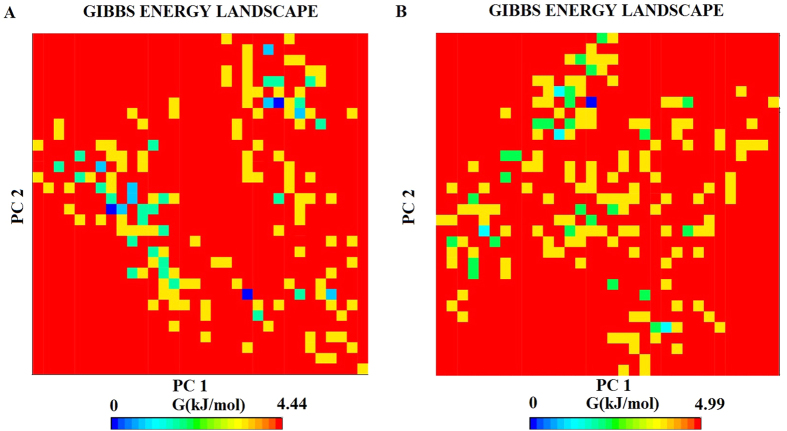
Projections of Free energy landscape of wild and mutant EZH2 conformational space onto PC1 and PC2 produced from PCA of Desmond trajectories. The free energy landscape along the first two principal components PC1 and PC2 for (**A**) wild type and (**B**) mutant, where dark blue indicates lowest energy configuration and red shows the highest energy configuration.

Free energy landscape was performed for all C-alpha of both the systems as seen in figure, where deep blue color indicated lowest energy based on the stability of the complex. The lowest energy for wild type system was found to be 0.08 kcal/mol while that of the mutant variant was 0.5 kcal/mol, indicating that mutation of residues Phe641 and Gly677 affected the overall conformational stability of the system (Fig. 12).

## Discussion

Breast and prostate cancer are one of the leading causes of death in female and male population respectively. This can be attributed to the fact that EZH2 subunit of the PRC2 complex, which plays an important role in the methylation of DNA resulting in the silencing of genes may sometimes be overexpressed, leading to silencing of those genes that are responsible for the suppression of cancer^7^. Commercially available compounds have been reported to act as potent inhibitors for this enzyme^8^.

The SET domain of EZH2 acts as the lysine methyltransferase for transferring methyl groups to the lysine side chain. The act of donating methyl groups to the side chain is dependent on SAM cofactor. The presence of this cofactor has been documented in other types of SET domains, however no evidence for the binding of substrates or SAM cofactors to the SET region of EZH2 has been found. In this study, the active site region of the 3D structure of EZH2 was modified to accommodate the binding of ligands as evidence of ligand-binding was not documented for the crystal structure. Molecular docking studies indicated that ligands bind readily to the newly modeled active site of the protein and showed interactions with important residues in the active site. Studies have also indicated that residues present in the active region of the protein are not directly involved in the process of methyltransferase^9^. However the presence of mutant residues, F641 and G677, have been shown to increase the trimethylation rate, and thus may be involved in the regulation of methyl groups added to the lysine residue^9^. Various computational approaches were undertaken in order to give a better insight on the binding mechanism of compounds to the active site of the protein, along with the effects of mutation of two specific amino acid residues, which were consistently linked with non-Hodgkin lymphoma, another form of cancer^10^. The top ranked compound STOCK1N-05528 proved to be an excellent candidate for the inhibition of the enzyme EZH2 for wild type and mutant alike.

Further, methods such as molecular dynamics simulation, principal component analysis and residue interaction network analysis were implemented for understanding the process of binding as well as to know the effects of mutation. These methods clearly explained the impact of mutation on the binding mechanism of compounds to the active site of the protein. Mutation of residues improved the overall binding energy by ~ −0.6 kcal/mol besides changing the landscape of the protein and the atomic interaction between the ligand-protein and the key residues. Findings from this study shows that mutation of two key residues near the active site affects the overall conformational landscape of the system, thus changing the interaction network.

## Methods

### Retrieval of target protein and its modification

Due to lack of evidence of a ligand binding site in the active region of the crystallographic structure, further analysis on the structure proved to be challenging. The 3 dimensional structure of EZH2 with PDB ID: 4MI5^11^ was retrieved from the protein databank (http://www.rcsb.org/pdb)^12^. Modeling of the SET region was carried out based on the X-ray crystal structure of hSET8 (PDB ID: 1ZKK^13^), the best sequence obtained via NCBI BLAST, using the Modeller graphical interface present in the Chimera package due to its high structural similarity with the EZH2 SET domain. The sequences from the two protein were aligned together to retain the structurally conserved regions using the complete amino acid sequence of EZH2 as the query. Alignment of the query sequence with the two aligned templates were done using pairwise alignment and the 3D structure of the template sequence, for which the structural part is missing, was generated using the skeletal framework of alpha carbon chain of the target structure. Two separate systems were created: (I) Wild type complex (II) F641 and G677 mutant complex. Mutation of F641 and G677 were done using Schrodinger Maestro, prior to the model refinement and energy minimization process. The amino acid residues to be mutated were replaced using the “mutate” option present in Maestro. To check the structure for conformational stability and steric clashes, the model was refined using the protein model refinement tool. From the output obtained via model refinement, the 3D structure with the best conformation, including rotatable bonds, was selected for further docking analysis and molecular dynamics studies. Preparation of the PDB structure was made using Maestro Protein Preparation Wizard, Schrodinger, LLC, New York, NY, 2014^14^.

### Ligand selection and preparation

InterBioScreen natural compound database (September 2015), containing more than 62000 compounds was downloaded for the structure based drug design. The ligands were prepared using ligprep and the best compound was selected based on the lowest energy with correct chirality. Calculation of absorption, distribution, metabolism, and excretion (ADME) properties^15^ and screening of ligands was done using Qikprop. Lipinski’s rule of five is drug-likeness refinement for the prediction of a chemical compound as an orally active drug based on its pharmacological and biological properties. Lipinski’s Rule of Five states that an orally active drug should:

1. Not have more than 5 hydrogen bond donors (OH and NH groups);
2. Not have more than 10 hydrogen bond acceptors (notably N and O);
3. Possess a molecular weight under 500 g/mol; and
4. Have a partition coefficient log P less than 5.

Lipinski rule of five was used to filter the compounds during virtual screening by the use of QikProp module of Schrodinger. QikProp bases its predictions on the full 3D molecular structure; unlike fragment-based approaches, QikProp can provide equally accurate results in predicting properties for molecules with novel scaffolds as for analogs of well-known drugs. Another rule, known as the rule of three or Jorgensen’s rule was used for predicting oral bioavailability. This rule was calculated using Schrodinger QikProp and is based in the Lipinski rule of five.

### Virtual screening and molecular docking

Virtual screening and molecular docking analysis of EZH2 with the best compounds were carried out by GLIDE, Schrodinger, LLC, New York, NY, 2014. The screening process of Glide includes three steps; High Throughput Virtual Screening (HTVS), Standard Precision (SP) and Extra precision (XP). In this research, XP docking was implemented. Conformations of compounds are generated in Glide internally which are then passed through a series of filters. In XP docking, compounds which display good scores for various interactions and avoid penalties are selected, thus removing false positives. The scoring function for the final docking results are given by the Glide G-score or the GlideScore, which can be represented as:


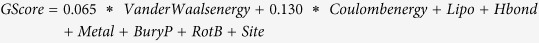


Where Lipo is a term favouring hydrophobic interactions, Metal for metal binding, BuryP for the buried polar group penalty, RotB as the penalty for freezing rotatable bonds and Site for polar interactions present in the active site. Reported inhibitors of the target protein like GSK126, EPZ005687, EL1 and UNC1999 were collected as reference molecules and were docked for both the wild and mutant grid for comparative analysis using GLIDE, Schrodinger, LLC, New York, NY, 2014.

### Molecular dynamics simulation

Molecular dynamics simulation for the top ranked complex was carried out using Desmond. Optimized Potentials for Liquid Simulations (OPLS) force-field was used for amino acid interaction, with simulation of water model by SPC (simple point charge) method. The addition of SPC water molecules (to cover the surface of the entire complex, 10 Å × 10 Å × 10 Å water box of orthorhombic dimensions was created) was carried out. For balancing the net charge, 2 Cl^−^ counter ions were added for neutralizing the system. Minimization of the system was made for 2000 iterations on a convergence threshold of 1 kcal/mol/Å and pre-equilibrated by using the inbuilt relaxation protocol. The whole system was subjected to 300 K for 50000 ps at NPT ensemble of MD simulation with a recording interval of 100 ps for total energy. Calculation of the root mean square deviation (RMSD) and potential energy were carried out to analyze the changes in structure and dynamics of the modelled protein^16^.

### Residue Interaction Network

Residue Interaction Network (RIN) Analysis is a technique that shows all the residues in a protein and how various interactions take place between in the form of a detailed network model. Each residue of a protein is represented as nodes and the interaction between pairs of residues is represented as edges. The crystal structure of both the wild type as well as the mutant protein were submitted in RING server^17^ for the construction of Cytoscape archive file. These Cytoscape archive files were then used for constructing the interactive residue interaction network (RIN) by using Cytoscape 3.2.1^18^.

### Visual Analysis of Residue Networks

Visualization of the network generated by the RING server was observed in Cytoscape using the plugin RINalyzer. The edges such as hydrogen bonds, salt bridges, and van der Waal interactions are labelled based on the type of interaction.

### Residue Interaction Network comparison

Alignment of the wild type complex with the mutant complex was done using RINalyzer alignment tool. Residues were selected based on the interaction with the ligand containing different nodes and edges. Dashed or dotted edges indicate the distinct interactions between the structures.

### Principal component analysis (PCA)

PCA is a standard tool in statistical mechanics used in order to determine the correlated motions of the residues to a set of linearly uncorrelated variables called principal components. This method is based on the construction of the covariance matrix of the coordinate fluctuations of the simulated proteins. The eigenvectors and eigenvalues are obtained by diagonalizing the covariance matrix, which provides information about correlated motions throughout the protein.

ProDy interface was used to perform PCA using normal mode wizard (NMW) of VMD^19^ for C-alpha atoms for both the systems. The PCA scatter plots were then generated using the xmgrace program (http://plasma-gate.weizmann.ac.il/Grace/).

### Free energy landscape (FEL)

Free energy landscape (FEL) describes the probability energy distribution as a function of one or more collective variable of the protein system which helps to visualize the stability of different conformations for a protein^20^. The stability of the protein is defined in terms of Gibb’s free energy. Gibb’s free energy is a function of the enthalpy and entropy of protein which analyzes the different conformational states that are important for protein’s structure-function correlation. Free energy values of backbone atoms of both wild and mutant systems were calculated using GROMACS^21^,^22^,^23^,^24^.

## Additional Information

**How to cite this article**: Aier, I. *et al*. Structural insights into conformational stability of both wild-type and mutant EZH2 receptor. *Sci. Rep*. **6**, 34984; doi: 10.1038/srep34984 (2016).

## Figures and Tables

**Table 1:** Docking analysis of EZH2 with top ranked screened compound and reported compounds with interacting residues.

Sl no.			*Compound ID*	*GLIDE G score*	*Interacting residues*
1	Wild type	Screened ligand	STOCK1N-05528 1, 3-Bis (1H-benzimidazol-2-yl) propan-1-amine	−11.6	Gln 648, Asn 668, Leu 666, Glu 740, Phe 665, Arg 685, Ile 739, Tyr 726, Phe 667, Val 737, Ala 733, Tyr 736, Gly 655, Asp 652, Ala 651, Val 764
2		Reported inhibitors	GSK126 1-[(2S)-butan-2-yl]-N-[(4,6-dimethyl-2-oxo-1H-pyridin-3-yl)methyl]-3-methyl-6-(6-piperazin-1-ylpyridin-3-yl)indole-4-carboxamide	−9.3	Phe 665, Ser 664, Tyr 726, Gly 655, Ala 651, Gln 648, Asp 652, Ala 733, Asn 668, Val 737, Leu 666, Tyr 736, Ile 739, Phe 686, Asn 788
3			EPZ005687 1-cyclopentyl-N-[(4,6-dimethyl-2-oxo-1H-pyridin-3-yl)methyl]-6-[4-(morpholin-4-ylmethyl)phenyl]indazole-4-carboxamide	−8.0	Gly 655, Tyr 658, Val 657, Arg 654, Ala 733, Tyr 736, Ala 651, Gln 648, Asn 668, Phe 665, Arg 685, Ile 739, Leu 666, Val 737, Ser 644, Asp 659
4			EPZ6438 N-[(4,6-dimethyl-2-oxo-1H-pyridin-3-yl)methyl]-3-[ethyl(oxan-4-yl)amino]-2-methyl-5-[4-(morpholin-4-ylmethyl)phenyl]benzamideZ6438	−7.3	Asn 688, Hie 689, Tyr 736, Val 737, Cyc 663, Ser 664, Asp 652, Gly 655, Gln 648, Val 674, Asn 668, Leu 666, Phe 665, Tyr 726, Ala 687, Arg 685, Phe 686
5			EL1 2-[(3R)-1-(5-bromothiophene-2-carbonyl)pyrrolidin-3-yl]oxy-4-[2-(methanesulfonamido)phenyl]-N-methylbenzamide	−7.0	Gly655, Arg 654, Leu 666, Asn 668, Val 674, Ala 733, Tyr 726, Ile 739, Val 737, Glu 738, Ala 651, Tyr 736
6			UNC1999 N-[(6-methyl-2-oxo-4-propyl-1H-pyridin-3-yl)methyl]-1-propan-2-yl-6-[6-(4-propan-2-ylpiperazin-1-yl)pyridin-3-yl]indazole-4-carboxamide	−6.5	Ala 651, Asp 652, Gln 648, Ala 733, Gln 730, Asp 732, Asn 668, Tyr 726, Asn 688, Hie 689, Glu 740, Leu 666, Phe 665, Ser 664
7	Mutant	Screened ligand	STOCK1N-05528 1, 3-Bis (1H-benzimidazol-2-yl) propan-1-amine	−12.2	Gly 655, Asp 652, Ala 651, Gln 648, Asn 668, Leu 666, Glu 740, Arg 727, Phe 665, Arg 685, Asn 688, Hie 689, Tyr 726, Tyr 728, Val 737, Phe 667, Val 674, Tyr 736
8		Reported inhibitors	GSK126 1-[(2S)-butan-2-yl]-N-[(4,6-dimethyl-2-oxo-1H-pyridin-3-yl)methyl]-3-methyl-6-(6-piperazin-1-ylpyridin-3-yl)indole-4-carboxamide	−10.4	Leu 666, Phe 665, Ser 664, Val 737, Tyr 736, Asn 668, Gln 648, Glu 730, Asp 652, Ile 739, Ala 687, Tyr 726, Asn 688
9			EPZ005687 1-cyclopentyl-N-[(4,6-dimethyl-2-oxo-1H-pyridin-3-yl)methyl]-6-[4-(morpholin-4-ylmethyl)phenyl]indazole-4-carboxamide	−11.2	His 689, Ile 739, Val 737, Tyr 726, Glu 740, Ala 733, Asp 732, Tyr 736, Asp 652, Gln 648, Ala 651, Gly 655, Arg 654, Asp 659, Ser 665, Leu 666
10			EPZ6438 N-[(4,6-dimethyl-2-oxo-1H-pyridin-3-yl)methyl]-3-[ethyl(oxan-4-yl)amino]-2-methyl-5-[4-(morpholin-4-ylmethyl)phenyl]benzamide38	−10.1	Ser 664, Cys 663, Met 662, Ala 687, Asn 668, His 689, Leu 666, Phe 686, Trp 624, Arg 685, Phe 665, Tyr 726, Val 737
11			EL1 2-[(3R)-1-(5-bromothiophene-2-carbonyl)pyrrolidin-3-yl]oxy-4-[2-(methanesulfonamido)phenyl]-N-methylbenzamide	−9.7	Asp 652, Ala 651, Tyr 736, Arg 654, Leu 666, Gln 648, Asn 668, Val 674, Ala 733, Tyr 726, Ile 739, Val 737, Gly 738
12			UNC1999 N-[(6-methyl-2-oxo-4-propyl-1H-pyridin-3-yl)methyl]-1-propan-2-yl-6-[6-(4-propan-2-ylpiperazin-1-yl)pyridin-3-yl]indazole-4-carboxamide	−8.5	Val 737, Phe 665, Ser 664, Tyr 661, Tyr 658, Val 657, Asp 659, Gly 655, Ala 651, Asp 732, Ala 733, Asn 668
